# The burden of cancer on primary and secondary health care services before and after cancer diagnosis in New South Wales, Australia

**DOI:** 10.1186/s12913-019-4280-1

**Published:** 2019-06-27

**Authors:** Stephen Morrell, Jane Young, David Roder

**Affiliations:** 1 Cancer Institute NSW, Level 9, 8 Central Avenue, Australian Technology Park, Sydney, NSW Australia; 2 Sydney School of Public Health, University of Sydney, Sydney, NSW Australia

**Keywords:** Cancer, Health system usage, General practitioners, Specialists, Hospital emergency departments

## Abstract

**Background:**

Primary and secondary healthcare service usage is assessed in the year before and following a cancer diagnosis, in cancer cases versus matched non-cancer controls in New South Wales (NSW), Australia over 2006–2012, for all invasive cancers collectively and for selected common sites: breast, prostate, colorectal and lung, and melanoma.

**Methods:**

The *45 and Up* cohort (*n* ≈267,000) was linked to NSW Cancer Register (NSWCR), Emergency Department Data Collection (EDDC) and Medical Benefits Schedule (MBS) data using probabilistic record linkage. First-ever malignant cancers diagnosed after enrolment in the *45 and Up* study comprised the study cases. Where possible, five controls were randomly selected per case from the *45 and Up* cohort, matched by sex and year of birth. Controls comprised those with no cancer recorded on the NSWCR. For each month in the year preceding and following the cancer diagnosis, general practitioner, specialist and specified hospital ED service use was compared between cases and controls using proportions, means, and odds ratios derived from conditional logistic regression.

**Results:**

Compared to controls, cases of all cancers combined had a significantly higher likelihood of GP and specialist consultation in the year leading up to diagnosis. This was most pronounced in the 3–4 months leading up diagnosis for all cancers, similarly for lung cancer (GPs and specialists) and melanoma (GPs), and colorectal cancer (specialists). Likelihood of a GP consultation remained significantly higher in cases than controls in the 12 months following diagnosis.

During most of the year preceding cancer diagnosis, the likelihood of specified ED presentations was also significantly higher in cases than controls for all cancers, and most pronounced in the 2–3 months before diagnosis. Excepting melanoma, the likelihood of specified ED presentations remained significantly elevated for most of the year following diagnosis for all cancers combined and for the selected cancers.

**Conclusions:**

People with cancer experience a higher use of primary and secondary healthcare services in the year preceding and following diagnosis, with GPs continuing to play a significant role post diagnosis. The higher likelihood of pre-diagnosis GP consultations among cancer cases requires further investigation, including whether signals might be derived to alert GPs to possibilities for earlier cancer detection.

## Background

Cancer is the single greatest cause of health burden in Australia, accounting for around 20% of the total disease burden [1]. Numerous studies have examined health-system usage around cancer diagnoses, including general practitioner and specialist services [2–9], hospital emergency department (ED) presentations [10, 11], and diagnostic testing [4]. Patterns of pre- and post-diagnosis follow-up by different components of the health system vary from country to country, and by cancer type.

Extent of GP and specialist involvement in cancer diagnosis and follow-up has already been explored: at a population level in Denmark for cancers overall, as monthly rates of GP and other health service use in the 12 months prior to and following a cancer diagnosis [4]; and by major cancer as a time-to-event study in the UK two years pre- and post-cancer diagnosis [5].

These results indicated the extent to which healthcare service use differed between cancer and non-cancer controls in Denmark and the UK, both before and following cancer diagnosis, enabling inferences of the potential burden of cancer on primary and secondary healthcare services.

Our aim is to determine usage patterns for New South Wales (NSW), Australia. In Australia the health system has two principal components. The first is a government sector, tax funded, and administered by national and jurisdictional governments. The second is a private sector subsidized through tax-funded universal health insurance and supplemented with elective private insurance. Most public or private health system treatment/consultation episodes are routinely recorded as cost or treatment items in health system databases at jurisdictional and/or national levels.

The present study is a more detailed replication of the Danish study [4], undertaken to determine differences in Australian health-system use by cancer cases compared with non-cancer controls before and after cancer diagnosis using cancer data from NSW, Australia. In addition to all cancers, we examine healthcare usage in relation to melanoma, and lung, colorectal, prostate and breast cancer.

### Hypotheses

Hypotheses were informed by results of the Danish study [4], namely:

- **H1:** The likelihood of GP use by cancer patients before and after the cancer diagnosis is significantly higher than in non-cancer controls.
- **H2:** Cancer patients have a significantly higher likelihood of using ED services (note: including outpatient day-only services and ED episodes leading to hospital admission) before and after the cancer diagnosis than for non-cancer controls.
- **H3:** The likelihood of specialist consultation use by cancer patients before and after cancer diagnosis is significantly higher than for non-cancer controls.

## Methods

### Data sources

The *45 and Up* cohort (*n* ≈267,000) was linked to NSW Cancer Register (NSWCR), Emergency Department Data Collection (EDDC), NSW Registrar of Births, Deaths and Marriages (RBDM) and Medical Benefits Schedule (MBS) data through the Centre for Health Record Linkage (CHeReL), using probabilistic record matching [12]. Probabilistic linkage attempts to maximise the likelihood of a correct record linkage when the details of some fields for linking may differ across different data sources. Linkage fields that don’t match differ in importance and a weight can be assigned to each to produce an overall likelihood of a correctly linked record. The weightings of the linking fields can also be changed to suit the particular populations and the purposes of a linkage.

The EDDC provided information on the frequency of designated outpatient presentations (i.e., those involving treatment or leading to a hospital admission). GP and consultant physician episodes were identified through MBS data, the central repository of Medicare-funded healthcare in Australia. Item codes in the MBS data used to identify GP attendances were ‘01’ to ‘51’, and for a specialist/consultant physician, were ‘99’ to ‘110’ and ‘112’ to ‘133’ [13]. Only first-ever cancers diagnosed after enrolment in the *45 and Up* study were considered as cases in this analysis. Controls comprised *45 and Up* cohort members with no cancer recorded in the NSWCR. Information on deaths was determined using NSW RBDM and NSWCR data.

All *45 and Up* survey participants gave permission for the record linkage [14], and the present study was approved by the NSW Population and Health Services Human Ethics Committee (Approval # HREC/14/CIPHS/60).

### Analysis

For each month pre- and post-cancer diagnosis, service episodes and proportions of subjects having designated services (i.e., GP or consultant physician/specialist episodes and specified hospital ED services) were compared between cases and five randomly selected non-cancer controls (where available) matched by sex and year of birth. Conditional logistic regression was used to estimate for cancer cases compared with controls in each month of the year pre- and post-cancer diagnosis, the odds ratios (ORs) of a GP or consultant physician episode and the ORs of specified hospital emergency department (ED) presentations. Mean monthly numbers of these encounters were also compared between cases and controls pre- and post-diagnosis and in the pre-diagnosis period, using t-tests. SAS ^*Ⓡ*^ version 9.4 was used for the analyses and plots.

**Table 1:** Mean monthly general practitioner, consultant/specialist episodes and hospital emergency department presentations for treatment or hospital admission in 12 months pre- and post-cancer diagnosis, major and all cancers diagnosed in *45 and Up* cohort, 2006–12

**Cancer**	Yes/No	General		Consultant/		ED		Admission	
		Practitioner		Specialist		treatment		from ED	
		Pre	Post	Pre	Post	Pre	Post	Pre	Post
Breast	Yes	0.40^x^	3.04^***^	0.05	0.10^***^	0.011	0.030^***^	0.008	0.024^***^
	No	0.38	0.38	0.05	0.05^*^	0.011	0.011	0.007	0.008^*^
Colorectal	Yes	0.61^xxx^	1.68^***^	0.09^xxx^	0.13^***^	0.018^xxx^	0.032^***^	0.026^xxx^	0.060^***^
	No	0.51	0.50	0.07	0.07^***^	0.014	0.014	0.011	0.013^**^
Lung	Yes	0.73^xxx^	2.54^***^	0.14^xxx^	0.15	0.026^xxx^	0.047^***^	0.037^xxx^	0.134^***^
	No	0.51	0.49^***^	0.07	0.07	0.013	0.014	0.011	0.014^***^
Melanoma	Yes	0.61^xxx^	1.05^***^	0.07^xxx^	0.07	0.012	0.018^***^	0.010	0.016^***^
	No	0.45	0.45	0.06	0.07^***^	0.011	0.013^**^	0.009	0.011^*^
Prostate	Yes	0.51^xxx^	2.11^***^	0.08^xxx^	0.11^***^	0.016^xxx^	0.022^***^	0.010	0.020^***^
	No	0.47	0.47	0.07	0.07^***^	0.012	0.013^*^	0.010	0.012^***^
All	Yes	0.59^xxx^	2.11^***^	0.08^xxx^	0.10^***^	0.018^xxx^	0.029^***^	0.020^xxx^	0.049^***^
	No	0.47	0.46^***^	0.06	0.07^***^	0.012	0.013^***^	0.010	0.012^***^

Significance (by t-test): post-diagnosis compared to pre-diagnosis (*); cancer compared to non-cancer, pre-diagnosis (x):

^***/xxx^p <.0001

^**/xx^.0001 ≤*p*≤.001

^*/x^.001 <*p*<.05

Deaths from cancer or other causes, whether obtained from either the NSWCR or NSW RBDM data, were used as the study endpoint. For example, if a death occurred on, say, May 5 2008 and this occurred in the 8th month following diagnosis of the relevant cancer case, then this record, whether a case or control, was excluded (censored) from all analyses of health system episodes occurring for months 9–12 following diagnosis. This is distinct from those people who are still alive who may have had no interactions with the health system, and whose events therefore were zero but still contributed to mean service-usage values and proportions.

## Results

### The study population

The final study group comprised 16,750 people diagnosed with cancer with no previously recorded cancer, diagnosed between 16 February 2006 and 26 May 2015, following their enrolment in the *45 and Up* study. These cases comprised 1999 patients with breast, 2077 with colorectal, 1235 with lung, 3960 with prostate, and 5409 with other cancers, plus 2070 with melanomas of the skin. A total of 76,827 controls was randomly selected after matching to cases by sex and year of birth. A small number of cases had 3 or 4 controls rather the planned 5 controls due to lack of relevant matching controls.

### Trends in service use following diagnosis (derived from Table 1)

In the 12 months post-diagnosis, mean monthly GP consultations, specialist consultations and hospital ED presentations resulting in treatment or admission increased considerably in cases compared with the 12 months pre-diagnosis. Compared with this pre-diagnostic period, the post-diagnostic increase among cases was ×3.6 (i.e., 3.6≈2.11÷0.59) for GP visits (*p* <0.001), ranging from ×1.7 for melanoma to ×7.6 for breast cancer. Increases in service utilisation in cases following diagnosis were lower at: (1) ×1.3 for consultant/specialist services (*p* <0.001) (ranging from ×1.0 for melanoma to ×2.0 for breast cancer); (2) ×1.6 for ED treatment (*p* <0.001) (ranging from ×1.4 for prostate cancer to ×2.7 for breast cancer); and (3) ×2.5 for ED admission (*p* <0.001) (ranging from ×1.6 for melanoma to ×3.6 for lung cancer). In general, increases in service utilisation were more marginal for controls, ranging from ×0.98 for GPs to ×1.20 for ED admission.

### Differences in service use between cases and controls (derived from Table 1)

In the 12 months prior to diagnosis, GP utilisation was significantly (*p* <0.001) higher in cases than controls at ×1.3 for all cases, and ranged from ×1.1 for breast and prostate cancers to ×1.4 for melanoma and lung cancers. The post-diagnosis elevation in GP utilisation was higher in cases than controls at ×4.6 (ranging from ×2.3 for melanoma to ×8.0 for breast cancer). Meanwhile, specialist utilisation was higher in cases than controls prior to diagnosis at ×1.3 (*p* <0.001) (ranging from ×1.0 for melanoma to ×2.0 for breast cancer) and post diagnosis at ×1.4 (ranging from ×1.0 for melanoma to ×2.1 for lung cancer). For ED treatment, the elevation was ×1.5 pre-diagnosis (*p* <0.001) (ranging from ×1.0 for breast cancer to ×2.0 for lung cancer) and post-diagnosis at ×2.2 (ranging from ×1.4 for melanoma to ×3.4 for lung cancer). ED admission data also showed elevated utilisation: pre-diagnosis for cases than controls at ×2.0 (*p* <0.001) (ranging from ×1.0 for prostate cancer to ×3.4 for lung cancer) and post-diagnosis for cases than controls at ×4.1 (ranging from ×1.5 for melanoma to ×9.6 for lung cancer).

### Trends of monthly service usage by cases and controls (Figs. 1 and 2)

**Fig. 1:**
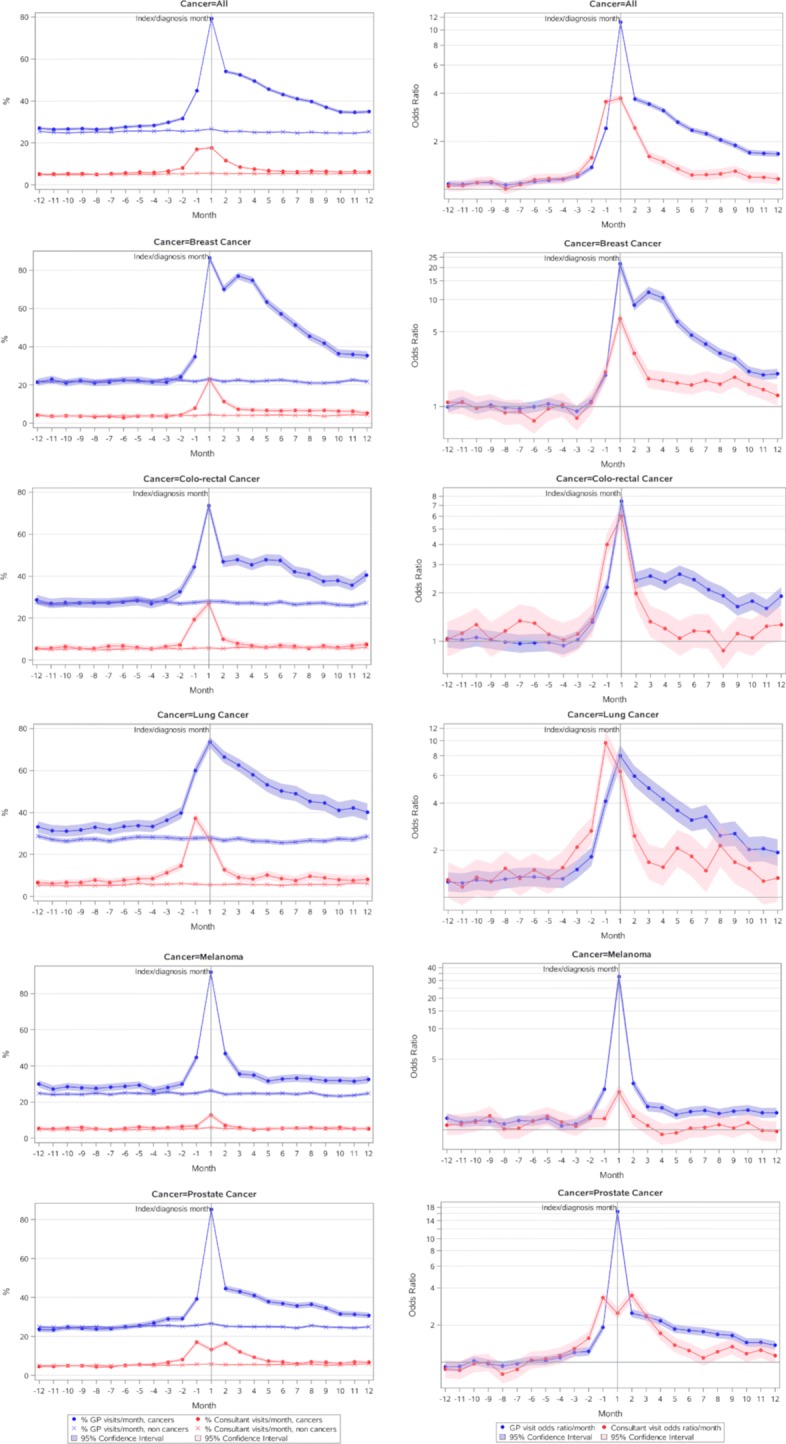
Proportions and odds ratios of general practitioner and consultant/specialist episode of care, 12 months pre- and post-cancer diagnosis, all cancers, melanomas, and breast, colorectal, lung and prostate cancers. Note: the index diagnosis month is labelled ‘1’ to indicate service episodes occurring in the month including the date of diagnosis, with ‘-1’, 2, etc, indicating services occurring in the months before and after the diagnosis month

**Fig. 2:**
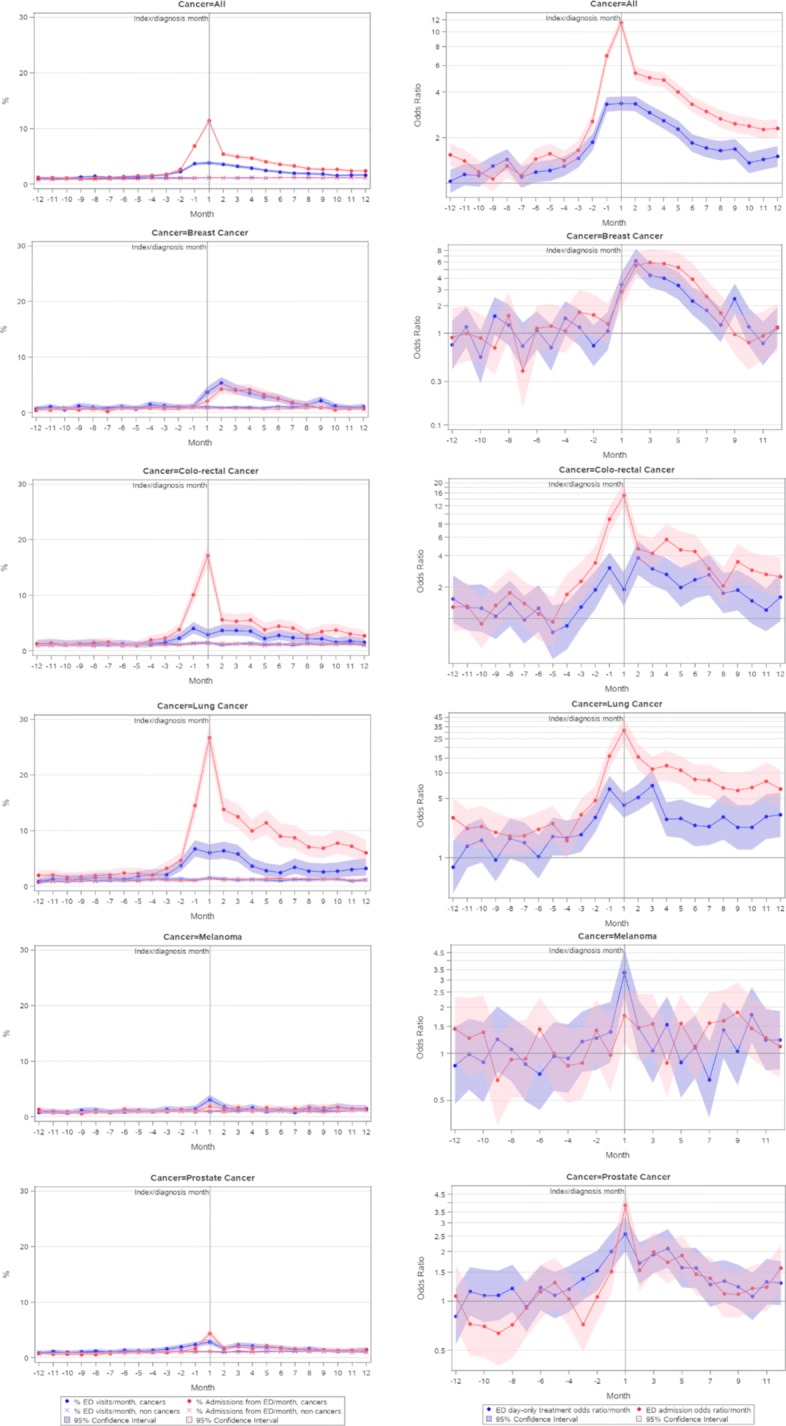
Proportions and odds ratios of hospital emergency department (ED) day visit and admission from ED, 12 months pre- and post-cancer diagnosis, all cancers, melanomas, and breast, colorectal, lung and prostate cancers. Note: the index diagnosis month is labelled ‘1’ to indicate service episodes occurring in the month including the date of diagnosis, with ‘-1’, 2, etc, indicating services occurring in the months before and after the diagnosis month

Trends of monthly health service usage, expressed as proportions of patients having at least one service use in each of the 12 months before and after the index cancer diagnosis, are presented in Figs. 1 and 2. Compared to controls, during the whole 12 months prior to the cancer diagnosis, GP consultations were significantly more likely in any given month for all cancers, and most pronounced in the 3–4 months prior to diagnosis with OR >11 in the diagnosis month (Fig. 1). A similar pattern to all cancers was also evident for melanoma and lung cancer. For the remaining selected cancers, an elevated likelihood of GP consultations occurred in the 2–4 months prior to diagnosis, and likelihood of a GP episode remained significantly higher for cancer cases than controls in the 12 months following diagnosis for all cancers and for each of the selected major cancers.

Specialist attendance was also significantly more likely prior to the cancer diagnosis in cancer cases than controls, for almost the whole 12 months pre-diagnosis for all cancers, peaking at OR ≈4 in the month of diagnosis and in the month prior (Fig. 1). The pattern was similar for lung and colorectal cancer. Melanoma also showed a higher likelihood of specialist consultation during the 12-month pre-diagnosis period, with the likelihood elevated in remaining cancers during the 2–3 months prior to diagnosis. The likelihood of cancer cases having specialist consultations declined relatively quickly after cancer diagnosis, but for cancers overall, and for breast, lung and prostate, this likelihood did not return to the levels seen in controls in the 12 months following diagnosis. Only for melanoma, and to a lesser extent colorectal cancer, did specialist consultations return to pre-diagnosis or control levels in the 12 months post diagnosis.

Eleven percent of all cancers had an ED presentation leading to hospital admission during the month of cancer diagnosis (Fig. 2). Of the selected major cancers, during the month of diagnosis, lung cancer had the highest proportion of admission from ED (27%), and colorectal cancer the second highest (17%). For all cancers, for most of the 12 months prior to diagnosis, the likelihood of a day-only treatment episode or a subsequent inpatient admission from an ED presentation was significantly higher in cancer cases than controls, which was most pronounced from 2–3 months before diagnosis. A similar pattern was evident for lung cancer; for colorectal cancer admission outcomes, and for prostate cancer day-only treatment episodes. Post-diagnosis hospital admissions from ED were significantly more likely than day-only outpatient treatment episodes for all cancers, and for all the selected cancers excepting melanoma and prostate cancer. The likelihood of an outpatient treatment or admission episode for all cancers, and major cancers excepting melanoma, remained significantly higher than for controls in most of the 12 months following diagnosis.

## Discussion

The present study shows that overall, GPs are more likely to be involved in the care of cancer patients in the months following than preceding cancer diagnosis, and at higher levels than for non-cancer controls in both periods. This confirms our a priori hypothesis H1 and is in accord with the GP episode patterns found in the Danish study [4]. By comparison, specialist consultations diminish quickly following cancer diagnosis, although remaining higher than for non-cancer controls. This is consistent with our a priori hypothesis H3, and is also similar to the patterns of diagnostic investigation episodes reported by the Danish study. It should be noted that a component of the decrease in specialist episodes after diagnosis may be artefactual due to non-billable episodes of specialist follow-up, hospital-based outpatient care, follow-up in chemotherapy clinics, for example. Nonetheless, cancer-specific differences in patterns of specialist care following diagnosis likely reflect true underlying cancer-related differences in intensity of specialist use.

The larger elevations in specialist consultations before than after cancer diagnosis likely reflect specialist involvement in the diagnostic work-up phase. To the extent that elevated specialist engagement occurs in the post-diagnostic phase, this may reflect involvement in monitoring and adjusting post-diagnostic treatments with systemic therapies and radiotherapy. This warrants confirmation and broader investigation. Although based on much smaller numbers, use of ED services, both for day-only procedures and preceding hospital admission, is elevated for most of the 12 months following cancer diagnosis, which is consistent with our a priori hypothesis H2 and accords with the Danish results. We suspect that some of this may reflect the provision of chemotherapy and other services beyond the scope of GPs, but this warrants confirmation and broader investigation. The greater use of GP services by cancer cases than controls, both before and after cancer diagnosis, is consistent with the Danish results. The pattern by month from 6–8 months pre-diagnosis and across the 12 months post diagnosis is suggestive of a cancer effect. This may represent an efficient use of the clinical workforce if GPs are performing some of the services effectively. Nonetheless, for cancers other than melanoma that require treatment beyond normal GP resources, higher rates of specialist involvement after diagnosis are the expectation and this is evident.

Higher GP consultations before cancer diagnosis have been reported in a Swedish study [15], and NSW data presented here show a significantly higher likelihood of GP consultation to apply to lung cancer and melanoma cases compared to controls for the entire 12 months prior to diagnosis (with age and sex controlled for). GP consultations become more elevated generally in the 2–3 months preceding diagnosis, including for cancers of the breast and colon/rectum that are included in national screening programs, and prostate cancer that is the subject of widespread opportunistic screening. These screened cancers show no significant differences from controls in GP and specialist consultations prior to the diagnostic work up in the 2–3 months preceding diagnosis.

This study has several limitations. The reasons for GP and other medical consultations are not recorded in MBS health insurance claims. Also, as noted above, some specialist consultations would be significantly under-recorded to the extent that they occurred in public hospital settings or were follow-up consultations without MBS payment. It is also possible that cancer cases may be at heightened risk of other diseases leading to medical consultations independent of cancer, due to common risk factors. Despite these weaknesses, the pattern of increases in consultations by month before and after diagnosis, and comparisons of consultation intensity after and before diagnosis within individual patients strongly implicates cancer as a dominant factor.

We believe this methodology could be further developed to complement routine analyses of hospital inpatient data and thus more fully indicate the burden of cancer on the health system. A similar approach may be useful to better determine the burden of other chronic diseases on the health system. A further opportunity requiring investigation is the prospect of using patterns of increased use of GP and other medical consultancies prior to diagnosis to reveal an increased risk of cancer [15], and to flag this to treating clinicians and patients through predictive models based on case-note recording. The extent that this may facilitate earlier diagnoses to enhance outcomes is worthy of further consideration.

## Conclusions

GPs continue to play a significant role post-diagnosis of cancer, whereas for most cancers, specialist consultations revert more quickly to levels expected in non-cancer patients.

The methodology used in this study could be employed to more fully describe the burden of cancer on the Australian health system. It may have similar applications for considering the burden of other chronic diseases.

## Data Availability

The data that support the findings of this study are available from the NSW Ministry of Health, the NSW Registrar of Births, Deaths and Marriages, the Australian Department of Health and Human Services, and the Sax Institute. Restrictions apply to the availability of these data, which were used under license for the current study, and so are not publicly available. Data however may be made available upon reasonable request and with permission of the above-named data custodians.

## Abbreviations

CHeReL: Centre for health record linkage
CR: Cancer registry
ED: Emergency department
EDDC: Emergency department data collection
GP: General practitioner
MBS: Medical benefits schedule
NSW: New South Wales
NSWCR: NSW cancer registry
OR: Odds ratio
RBDM: Registrar of births, deaths and marriages
